# Exploring the immune microenvironment of osteosarcoma through T cell exhaustion-associated gene expression: a study on prognosis prediction

**DOI:** 10.3389/fimmu.2023.1265098

**Published:** 2023-12-15

**Authors:** Junchao Zhu, Jinghong Yuan, Shahrzad Arya, Zhi Du, Xijuan Liu, Jingyu Jia

**Affiliations:** ^1^ Department of Orthopedics, The Second Affiliated Hospital of Nanchang University, Nanchang, Jiangxi, China; ^2^ Division of Surgical Oncology, Department of Surgery, Massachusetts General Hospital, Harvard Medical School, Boston, MA, United States; ^3^ Department of Pediatrics, The Second Affiliated Hospital of Nanchang University, Nanchang, Jiangxi, China

**Keywords:** osteosarcoma, T-cell exhaustion, tumor microenvironment, prognosis, gbp2

## Abstract

**Background:**

Osteosarcoma is a highly aggressive type of bone cancer with a poor prognosis. In the tumor immune microenvironment, T-cell exhaustion can occur due to various factors, leading to reduced tumor-killing ability. The purpose of this study was to construct a prognostic model based on T-cell exhaustion-associated genes in osteosarcoma.

**Methods:**

Patient data for osteosarcoma were retrieved from the TARGET and GEO databases. Consensus clustering was employed to identify two novel molecular subgroups. The dissimilarities in the tumor immune microenvironment between these subgroups were evaluated using the “xCell” algorithm. GO and KEGG analyses were conducted to elucidate the underlying mechanisms of gene expression. Predictive risk models were constructed using the least absolute shrinkage and selection operator algorithm and Cox regression analysis. To validate the prognostic significance of the risk gene expression model at the protein level, immunohistochemistry assays were performed on osteosarcoma patient samples. Subsequently, functional analysis of the key risk gene was carried out through *in vitro* experimentation.

**Results:**

Four gene expression signatures (PLEKHO2, GBP2, MPP1, and VSIG4) linked to osteosarcoma prognosis were identified within the TARGET-osteosarcoma cohort, categorizing patients into two subgroups. The resulting prognostic model showed strong predictive capability, with area under the receiver operating characteristic curve (AUC) values of 0.728/0.740, 0.781/0.658, and 0.788/0.642 for 1, 3, and 5-year survival in both training and validation datasets. Notably, patients in the low-risk group had significantly higher stromal, immune, and ESTIMATE scores compared to high-risk counterparts. Additionally, a nomogram was developed, exhibiting high accuracy in predicting the survival outcome of osteosarcoma patients. Immunohistochemistry, Kaplan-Meier, and time-dependent AUC analyses consistently supported the prognostic value of the risk model within our osteosarcoma patient cohort. *In vitro* experiments provided additional validation by demonstrating that the downregulation of GBP2 promoted the proliferation, migration, and invasion of osteosarcoma cells while inhibiting apoptosis.

**Conclusion:**

The current study established a prognostic signature associated with TEX-related genes and elucidated the impact of the pivotal gene GBP2 on osteosarcoma cells via *in vitro* experiments. Consequently, it introduces a fresh outlook for clinical prognosis prediction and sets the groundwork for targeted therapy investigations in osteosarcoma.

## 1 Introduction

Osteosarcoma, a malignant bone tumor commonly diagnosed in children and adolescents, poses a significant challenge due to its high metastatic rate and poor survival outcomes. Accounting for 56% of all primary malignant bone tumors, osteosarcoma remains one of the most aggressive forms of bone cancer (1, 2). Despite extensive efforts to improve prognosis, the 5-year survival rate for patients with metastatic osteosarcoma remains below 30% (3, 4). The complex molecular mechanisms and genomic instability associated with osteosarcoma have been identified as major contributors to its unfavorable prognosis (5).

T-cell exhaustion (TEX), a state of cellular dysfunction resulting from chronic infection or cancer, has emerged as a critical factor in tumor-related immune dysfunction. TEX is characterized by the upregulation of inhibitory receptors like programmed cell death 1 (PD-1), cytotoxic T lymphocyte-associated antigen 4 (CTLA-4), and T cell immunoglobulin domain and mucin domain-3 (TIM-3) on T cells, impairing their function (6–9). Studies have implicated TEX, particularly CD8+ T cell exhaustion, in tumor immune evasion (10–12). In an osteosarcoma xenograft model, inhibiting TIM-3 led to suppressed tumor growth and enhanced functional activation of CD8+ T cells within the tumor (13). Alleviating TEX has thus become a promising focus in cancer immuno-therapy research (14). However, the establishment of a reliable prognostic signature incorporating TEX-associated genes specific to osteosarcoma remains an unmet need. By modulating immune responses in the tumor microenvironment through strategies like immune checkpoint blockade or immune cell-based therapies, there exists potential for augmenting anti-tumor immune responses and improving outcomes in high-risk osteosarcoma patients.

In this study, we aimed to develop an osteosarcoma prognostic model centered around TEX-associated genes and explore its correlation with immune status. Our findings not only shed light on novel approaches for understanding the treatment and prognosis of osteosarcoma but also pave the way for innovative interventions targeting immune evasion mechanisms.

## 2 Materials and methods

### 2.1 Data collection

**Figure 1:**
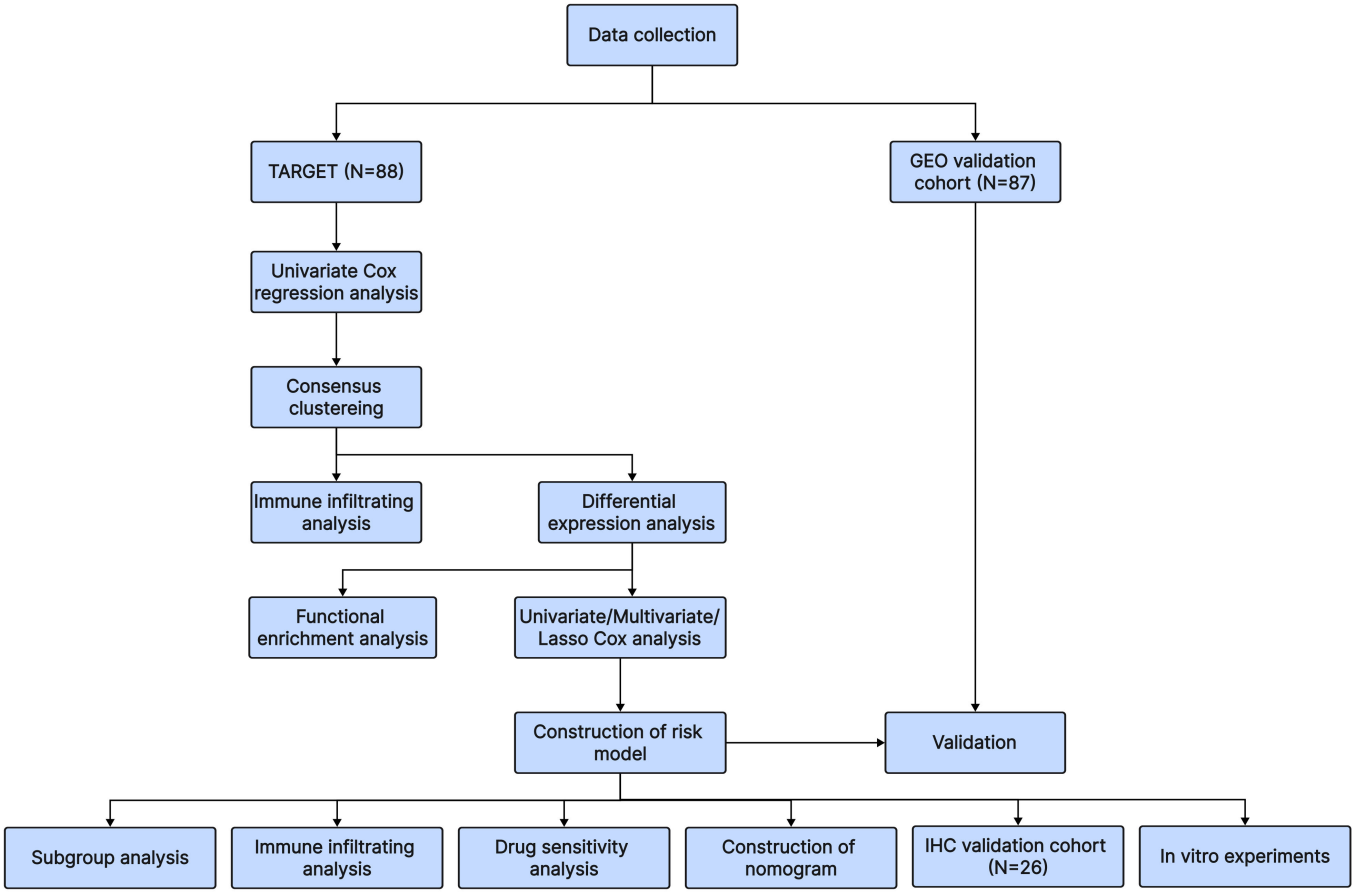
The flowchart of our research process.

Osteosarcoma patients in the training cohort were identified using clinical information and RNA sequencing data from the Therapeutically Applicable Research to Generate Effective Treatments (TARGET) database, which contained 88 samples. We merged the sequencing data and clinical data from GSE21257 and GSE16091, a total of 87 samples, to construct the validation cohort using material from the Gene Expression Omnibus (GEO, https://www.ncbi.nlm.nih.gov/geo/) database. 40 T-cell exhaustion-associated genes were obtained from the article by Zhang Z et al. (15). The overall workflow in this study is presented in **Figure 1**.

### 2.2 Identification of molecular subgroups

After data collection and processing, it was found that six genes (WAS, TLL1, SP140, PRKD2, PRF1, LOX) were associated with the prognosis of osteosarcoma by univariate Cox regression analysis. The “Consensus ClusterPlus” R package was used to perform the cluster analysis based on the expression matrix of the six genes.

### 2.3 Immune analysis

To compare the differences in immune infiltration between the two clusters, we selected the “xCell” algorithm to calculate the immune cell infiltration abundance and immune scores based on the gene expression of the samples in the TARGET training cohort using the “IOBR” R package. The “CIBERSORT” algorithm was utilized to ascertain the correlation between genes implicated in modeling and the infiltration patterns of immune cells.

### 2.4 Differentially expressed genes and functional analysis

Using the “Limma” R package, differentially expressed genes (DEGs) between the two clusters were found. The “ClusterProfiler” R package was used to conduct functional studies, including Gene Ontology (GO) and Kyoto Encyclopedia of Genes and Genomes (KEGG) analyses. Meanwhile, Genome Enrichment Analysis (GSEA) was performed for the purpose of verifying functional differences between the two clusters. In addition, we obtained gene sets of Ferroptosis (16), Pyroptosis (17), Necroptosis (18), and Immuno-genic cell death (19) from previous studies. After that, these genes were analyzed by comparing the expression between the two clusters.

### 2.5 Establishment of the risk model

2697 co-expressed genes of 40 TEX-associated genes were found using the Stat R package, and 337 genes were screened by crossing DEGs between the two clusters. Among the 337 genes, Univariate and multivariate Cox regression analyses were used to identify 72 genes associated with prognosis. After that, the “glmnet” R package was used to run the Lasso-Cox regression analysis for 72 genes. In the end, we obtained four genes by setting lambda equal to 0.1402. The risk score of each patient is as follows: Risk score=-0.2182 × expression value of PLEKHO2 - 0.0392 × expression value of VSIG4 - 0.0022 × expression value of MPP1 - 0.0440 × expression value of GBP2. Furthermore, we used the STRING database (https://cn.string-db.org/) to present the interactions of the four genes used to establish the model and 40 T-cell exhaustion-associated genes at the protein level and visualized in Cytoscape version 3.9.1.

### 2.6 Evaluation and verification of the risk model

Using the above formula, we calculated the risk scores of each patient and divided them into high-risk and low-risk groups. The “surviminer” R package was used to plot the KM curves to compare survival between different groups. The “pROC” R package was used to plot the time-related ROC curve. The “rms” R package was used to build and verify the nomograph model and draw the calibration curve. The “ggplot2” R package was used to create the risk score and survival status distribution map.

### 2.7 Drug sensitivity analysis

The sensitivity score for each small molecule compound was computed for individual patients in both the high-risk and low-risk groups using the “oncoPredict” package.

### 2.8 scRNA-Seq data processing and analysis

Single-cell RNA sequencing (scRNA-seq) data was retrieved from the GSE152048 and GSE162454 datasets housed within the GEO database, encompassing a collective of 17 osteosarcoma samples. The standardized data underwent preprocessing via the “seurat” package (version 4.0). Rigorous quality control measures were applied, excluding cells with fewer than 300 or more than 2000 detected genes. To mitigate batch effects, the “CAA” package was employed. Post-filtering, a total of 179,499 cells remained for subsequent analyses. The identification of sample clusters was accomplished using the FindClusters function (resolution = 0.6). Dimensionality reduction techniques, namely t-distributed stochastic neighbor embedding (t-SNE) and Uniform Manifold Approximation and Projection (UMAP), were employed. AUCell analysis was conducted utilizing the R package AUCell (version 1.20.5). Additionally, we obtained the CD8+ exhausted T cell-related gene set (GSE9650_EXHAUSTED_vs_MEMORY_CD8_TCELL_UP. v2023.2.Hs.gmt) from the GSEA database to compute AUCell scores, specifically focusing on exhausted CD8 T cells within each cell population.

### 2.9 Immunohistochemistry analysis

A total of 26 osteosarcoma specimens, collected from February 2012 to October 2016, were obtained from the Second Affiliated Hospital of Nanchang University to serve as an external validation cohort. These specimens were formalin-fixed and paraffin-embedded blocks. To assess the predictive capability of the risk model for deter-mining vitality, immunohistochemical analysis was conducted on paraffin sections using PLEKHO2 (1:200, Proteintech, Cat. No 21356-1-AP, China), GBP2 (1:200, Proteintech, Cat. No 11854-1-AP, China), MPP1 (1:100, Affinity Biosciences, Cat. No AF9115, China), and VSIG4 (1:100, Affinity Biosciences, Cat. No DF14591, China) antibodies following standardized protocols. The expression levels of PLEKHO2, GBP2, MPP1, and VSIG4 were evaluated using H-scores. The research that involved human subjects underwent review and received approval from the Ethics Committee of The Second Affiliated Hospital of Nanchang University [Review (2020) No. (086)].

### 2.10 Cell lines and transfection

The human osteosarcoma cell line HOS and SaOS-2 were obtained from Wuhan Procell Life Science & Technology Co., Ltd and cultured in a 5% CO_2_ incubator at 37°C. To knockdown the expression of GBP2 (ENSG00000162645; GeneID: 2634), siRNA (RiboBio, Cat. No siG000002634A-1-5, China) was synthesized with the following sequence: 5′-GAGCCTCATTGATAACACT-3′. The control group cells were transfected with negative control siRNA (si-NC, RiboBio, Cat. No siN0000001-1-5, China). The riboFECT CP Transfection Kit (RiboBio, Cat. No C10511-05, China) was used for siRNA transfection.

### 2.11 RT-qPCR

Total RNA was extracted from the cells using the Trizol reagent RNA extraction kit (Invitrogen, Cat. No 15596026, USA) following the provided instructions. Reverse transcription of RNA was performed using the PrimeScript RT Master Mix Kit (Takara, Cat. No RR036B, Japanp). The specific primers and a real-time PCR kit (Takara, Cat. No RR820A, Japan) were used for quantitative PCR. The following primer sequences were used for qRT-PCR: GBP2 forward primer: 5′-GATTGGCCCGCT CCTAAGAA-3′, reverse primer: 5′-TTGACGTAGGTCAGCACCAG-3’, and GAPDH forward primer: 5′-GGAAATCCCATCACCATCTTC-3′, reverse primer: 5′-TGGAC TCCACGAC-GTACTCAG-3′. The relative expression level of RNA was analyzed using the 2^(-ΔΔCt) method.

### 2.12 Western blotting

Proteins were extracted from cells using RIPA lysis buffer (Beyotime, Cat. No P0013, China). Equal amounts of protein were separated by 10% SDS-PAGE gel electrophoresis and then transferred onto a PVDF membrane (BioRad, Cat. No 1620177, USA). The PVDF membrane was blocked with 5% skim milk and incubated overnight at 4°C with primary antibodies against GBP2 (1:1000, Proteintech, Cat. No 11854-1-AP, China) and GAPDH (1:5000, Proteintech, Cat. No 60004-1-Ig, China). After washing with TBST, the membrane was incubated with a secondary antibody (1:2000, Proteintech, Cat. No SA00001-1/SA00001-2, China) for 1 hour at room temperature. The chemiluminescence of proteins was detected using an ECL chemiluminescence kit (UElandy, Cat. No S6009M, China) and analyzed using ImageJ software.

### 2.13 Cell proliferation assays

Cell proliferation was assessed using the cell counting kit-8 (CCK-8) obtained from TransGen Biotech Co., Ltd (Cat. No FC101-02). Osteosarcoma cells were seeded at a density of 3000 cells per well in a 96-well plate. After the cells reached confluence, they were transfected with si-GBP2 and si-NC. At 0, 24, 48, and 72 hours, 10 µL of CCK-8 reagent was added to each well. After incubating for an additional 2 hours, the absorbance at 450 nm was measured to determine cell viability.

### 2.14 Transwell assays

For cell migration and invasion assays, Transwell chambers (Corning, Cat. No 3470, USA) were prepared with either uncoated or matrigel-coated membranes. The transfected osteosarcoma cells with si-GBP2 and si-NC were respectively trypsinized, collected, and suspended in serum-free medium at a density of 1×10^5^ cells/ml. Subsequently, 200 µL of the cell suspension was added to the upper chamber, while 600 µL of medium containing 20% FBS was added to the lower chamber. Following a 24-hour incubation period, cells in the lower chamber were fixed with 4% formaldehyde for 20 minutes and stained with 0.5% crystal violet for 15 minutes. The number of cells was counted in several randomly selected areas under an Olympus light microscope.

### 2.15 Wound healing assays

The transfected osteosarcoma cells with si-GBP2 and si-NC were respectively seeded onto six-well culture plates. When the cells reached approximately 90% confluency, a sterile pipette tip was used to create a scratch or gap on the cell monolayer. The cells were then cultured in serum-free medium for 12 hours. The gaps at 0 hours and 12 hours were observed and photographed under an inverted microscope.

### 2.16 Apoptosis detection

Apoptosis in osteosarcoma cells was assessed using the FITC Annexin V apoptosis detection kit (BD Biosciences, Cat. No 559763, USA) and a flow cytometer. According to the manufacturer’s instructions, the transfected osteosarcoma cells with si-GBP2 and si-NC were respectively collected and stained. Propidium iodide and An-nexin V-fluorescein-isothiocyanate stains were used to measure late and early apoptosis. The data were analyzed using FlowJo software.

### 2.17 Statistical analysis

The bioinformatics section underwent statistical analysis utilizing R version 4.0.3. KM survival analysis was executed employing the log-rank test, while comparisons between the two groups were conducted using the Wilcoxon rank sum test. In parallel, the statistical analysis for the *in vitro* experiments was conducted through GraphPad Prism version 8.0.1, where paired t-tests were employed for evaluation. A significance threshold of *p* < 0.05 was set for determining statistical significance (ns: *p* > 0.05; *: *p* < 0.05; **: *p* < 0.01; ***: *p* < 0.001, **** *p* < 0.0001).

## 3 Results

### 3.1 Identification of two molecular subtypes

**Figure 2:**
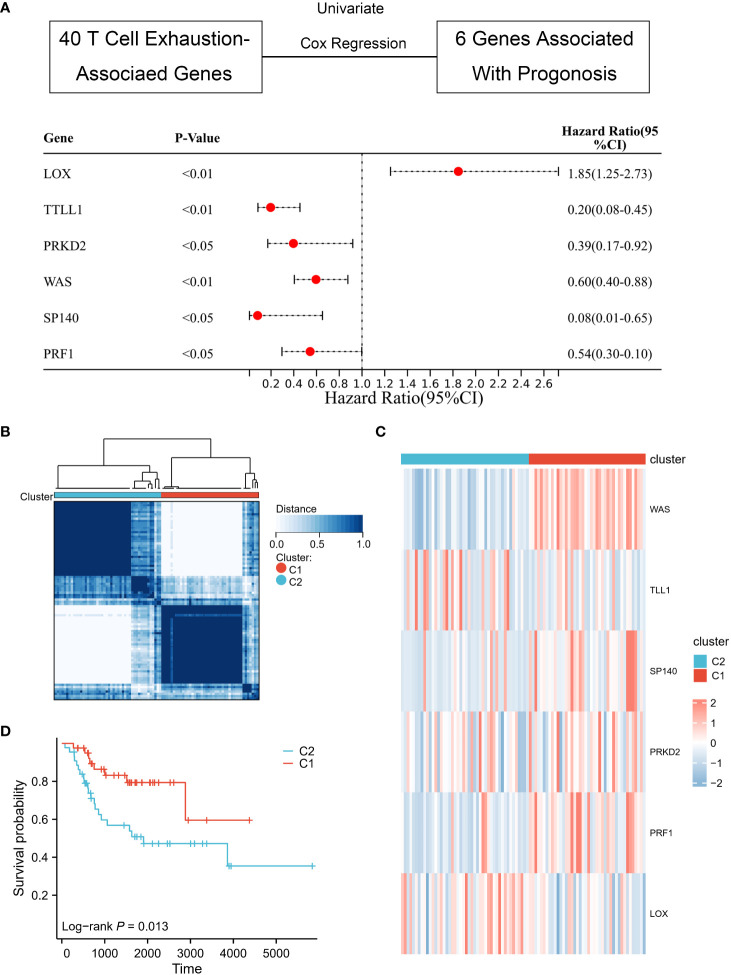
Identification of TEX-associated subtypes by consensus clustering. **(A)** Univariate Cox analysis revealed the presence of six genes that exhibit prognostic significance in relation to TEX-associated genes. **(B)** The heatmap represents the consensus clustering solution (k = 2) applied to the expression patterns of the aforementioned six genes across 88 osteosarcoma samples. **(C)** The heatmap visually represents the gene expression levels of the six TEX-associated genes within two distinct subgroups, denoted as C1 and C2. **(D)** The Kaplan-Meier survival curve effectively demonstrates that patients classified in the high-risk group (C2) exhibited a substantially lower overall survival rate compared to those in the low-risk group (C1).

Consensus clustering was employed to divide 42 patients into Cluster 1 and 46 patients into Cluster 2, based on the six genes that were screened and analyzed through univariate Cox regression analysis (**Figures 2A, B**). The heat map (**Figure 2C**) illustrated the expression profile of the TEX-associated genes in the two clusters and indicated that there were significant differences in their expression levels. Furthermore, patients in Cluster 1 displayed a superior overall survival rate compared to those in Cluster 2 (**Figure 2D**), suggesting that patients classified into the two subgroups based on TEX-associated genes exhibit divergent prognostic survival.

### 3.2 Immune difference between two subtypes

**Figure 3:**
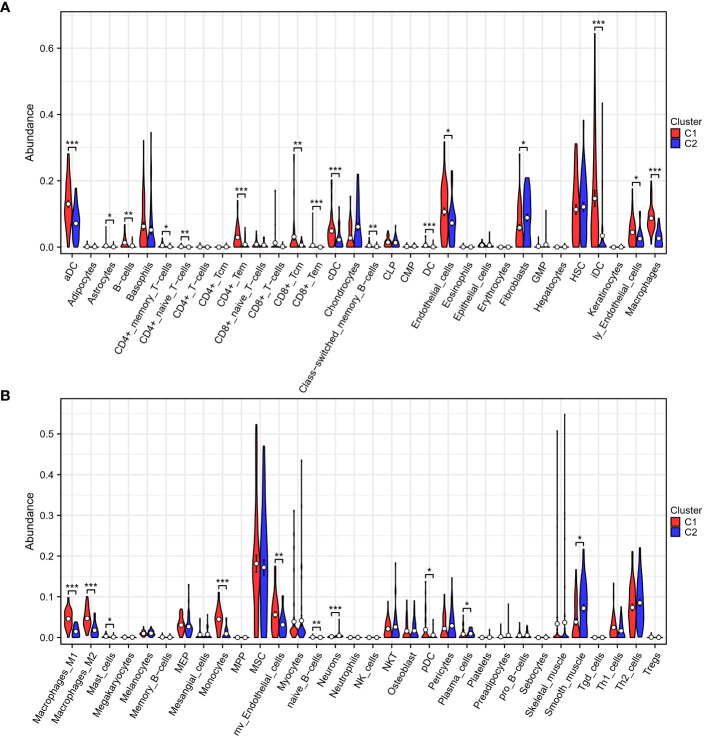
Immune analysis of two subgroups. **(A, B)** The xCell algorithm reveals a pronounced upregulation of CD4+ T cells, CD8+ T cells, dendritic cells, and macrophages in the low-risk group (C1), indicating an active and enhanced immune response within their tumor microenvironment. *p < 0.05, **p < 0.01, ***p < 0.001.

To delve further into the immune differences between the two clusters, the xCell algorithm was utilized to assess the enrichment difference of 64 immune and stromal cell types. These results were visualized as boxplots (**Figures 3A, B**) and indicated that patients classified into the two subgroups based on TEX-associated genes possess differing immune statuses.

### 3.3 DEGs and functional analysis

**Figure 4:**
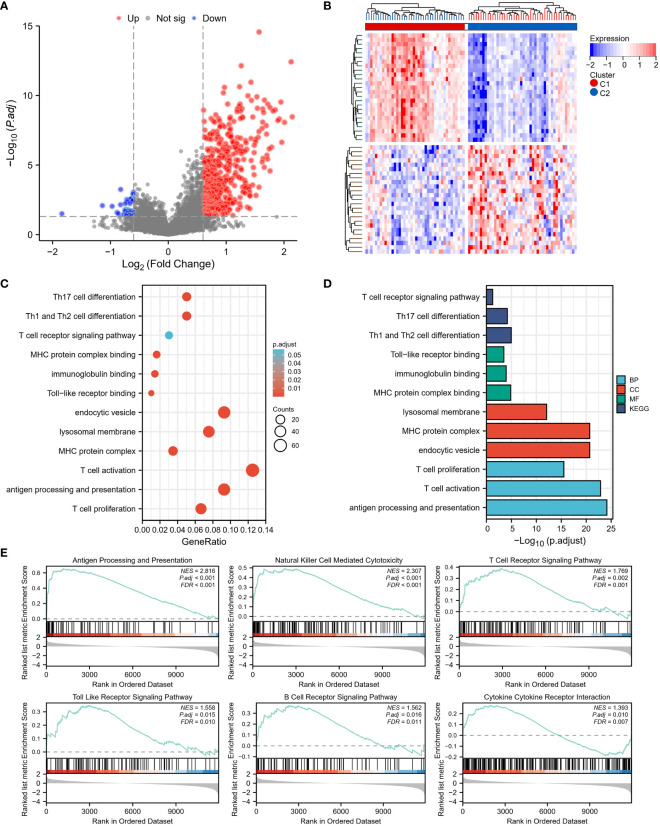
Differentially expressed genes (DEGs) and underlying signal pathways in different subtypes were identified. **(A)** The volcano plot showed the DEGs between the two subgroups. **(B)** The heatmap showed the DEGs expression in two subgroups. **(C, D)** The GO and KEGG analyses indicate that there is the enrichment of the proliferation and activity of T cells, antigen presentation, T cell receptor signaling, Toll-like receptor (TLR) signaling, and B cell receptor signaling pathway. **(E)** GSEA analysis determined the significant suppression of crucial signaling pathways involved in T cell receptor signaling, Toll-like receptor (TLR) signaling, B cell receptor signaling, antigen presentation, and cytokine receptor interaction in the high-risk group (C2).

To examine the mechanisms by which TEX-associated genes influence the prognosis of osteosarcoma patients, DEGs between the two clusters were analyzed and functional analysis was performed. Results showed that Cluster 1 had 547 DEGs in total, with 512 genes upregulated and 45 genes downregulated (**Figures 4A, B**). GO enrichment analysis revealed that the DEGs were largely enriched in immune-related terms such as antigen processing and presentation, T cell activation, and T cell proliferation. Similarly, KEGG enrichment analysis indicated that the DEGs were enriched in immune-related pathways, including Th1 and Th2 cell differentiation, Th17 cell differentiation, and T cell receptor signaling (**Figures 4C, D**). The results of GSEA analysis also showed that antigen processing and presentation, natural killer cell-mediated cytotoxicity, T cell receptor signaling pathway, Toll-like receptor signaling pathway, B cell receptor signaling pathway, and cytokine receptor interaction were more highly expressed in Cluster 1 (**Figure 4E**). These findings suggest that TEX-associated genes are closely linked to immune dysfunction, which might contribute to the poor prognosis of osteosarcoma patients.

**Figure 5:**
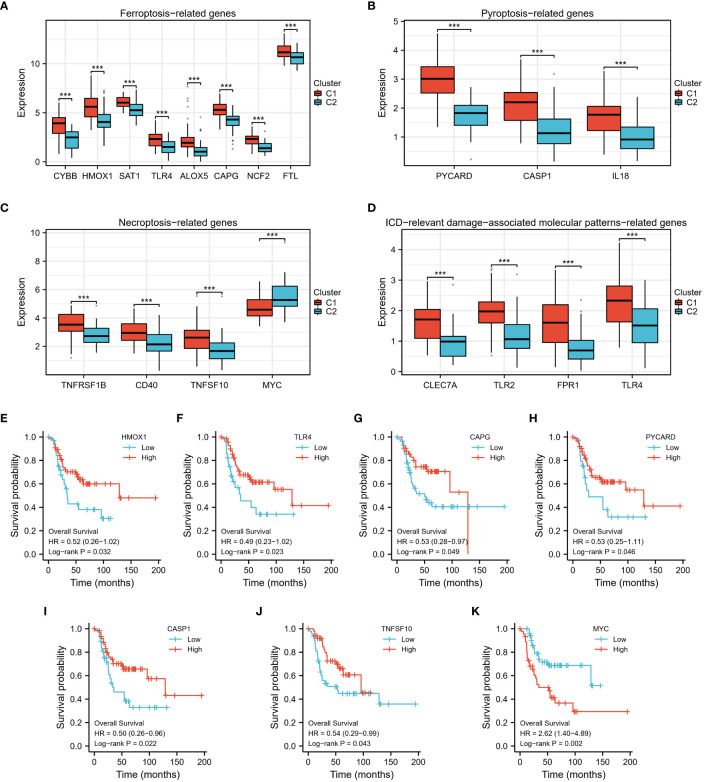
The differential expression of 4 types of cell death phenotype-related genes. **(A–D)** In the high-risk group (C2), the expression levels of numerous cell death phenotype-related genes, such as fer-roptosis-related genes, pyroptosis-related genes, necroptosis-related genes, and genes related to immunogenic cell death (ICD)-relevant damage-associated molecular patterns, were found to be significantly lower. **(E–K)** Kaplan Meier analysis demonstrated a significant difference in prog-nosis between high and low-expression groups of these genes. ***p < 0.001.

In addition, we analyzed the differential expression of ferroptosis-, pyroptosis-, necroptosis-, and immunogenic cell death (ICD)-related genes between the two clusters, and found that almost all of these cell death phenotype-related genes were expressed at higher levels in Cluster 1 compared to Cluster 2 (**Figures 5A–D**). KM analysis showed a significant correlation between HMOX1, TLR4, CAPG, PYCARD, CASP1, TNFSF10, MYC, and the prognostic survival of patients (**Figures 5E–K**). These results suggest that TEX-associated genes might be associated with the expression of cell death phenotype-related genes, leading to different prognoses for patients.

### 3.4 Establishment of prognostic signature

**Figure 6:**
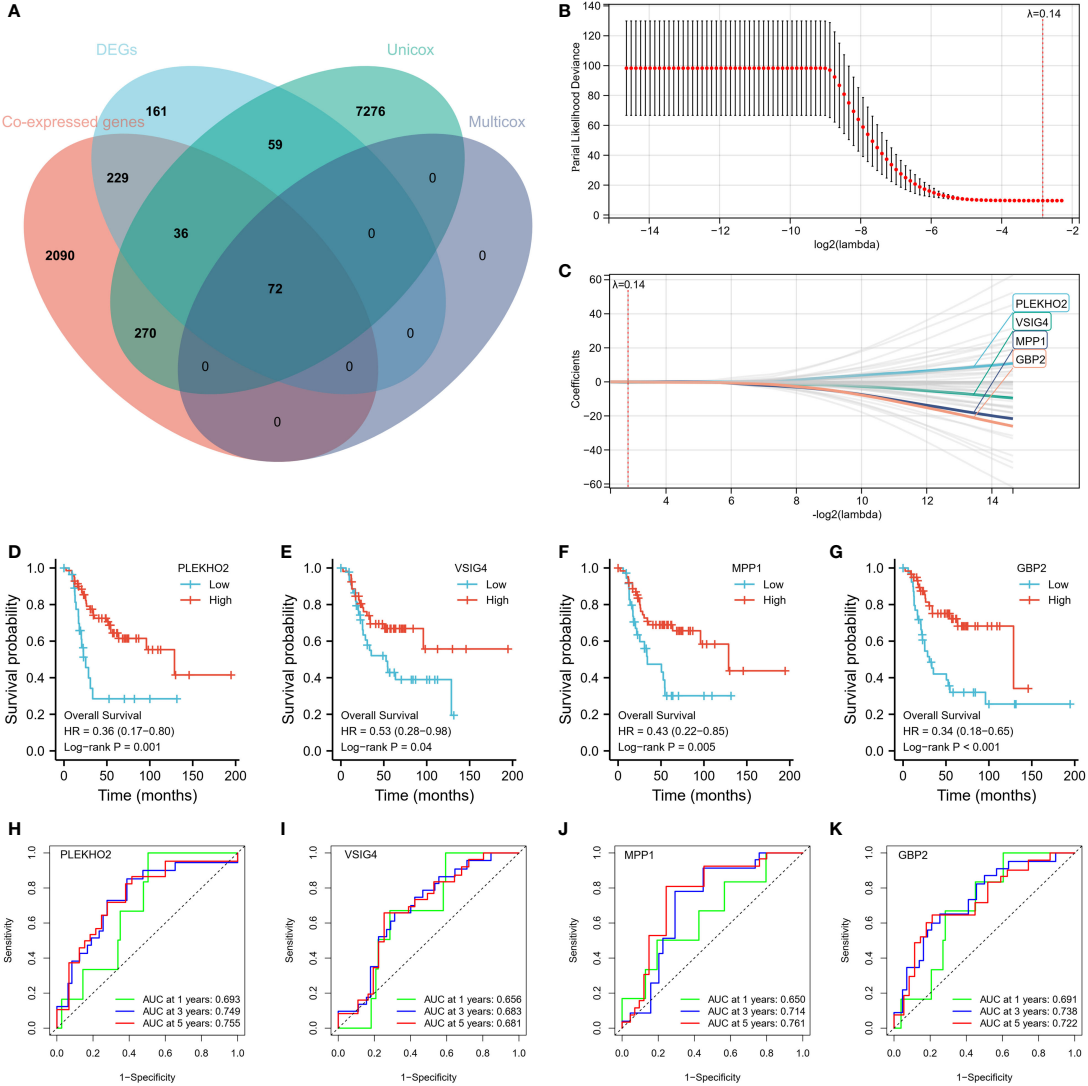
Construction of 4-gene Risk Signature. **(A)** A total of 72 genes were identified as prognostically relevant, as depicted in the Venn diagram. **(B, C)** LASSO analysis was performed with a minimal lambda value. The model achieved its highest stability and accuracy when lambda was set to 0.14. **(D–G)** Kaplan-Meier survival curves were successfully established for a model consisting of four genes: PLEKHO2, VSIG4, MPP1, and GBP2. **(H–K)** The significance of these genes as prognostic indicators was confirmed through Kaplan-Meier analysis and ROC curves.

**Figure 7:**
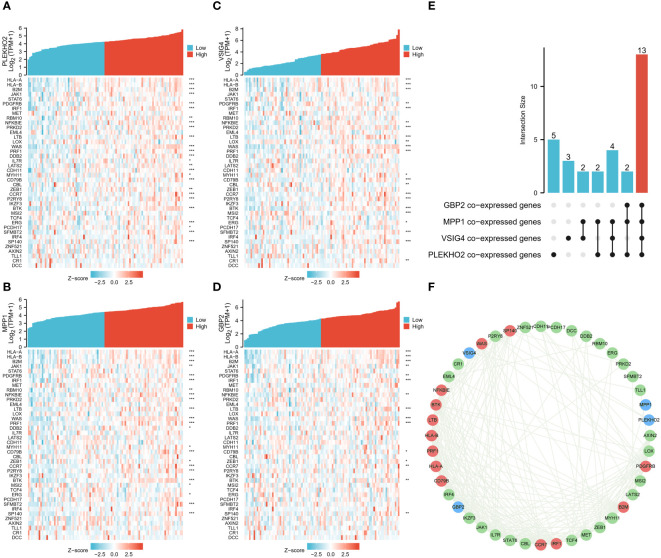
Correlation analysis of the four genes in the model and TEX-associated genes. **(A–D)** Gene expression-related heat maps of the four genes in the model and TEX-associated genes. **(E)** The upset plot depicted the overlapping genes among four groups of TEX-associated genes that were co-expressed with the modeling genes (PLEKHO2, VSIG4, MPP1, GBP2). **(F)** The interactions between the four modeling genes and 40 TEX-associated genes at the protein level were presented using the STRING database. The modeling genes were indicated in blue, while the TEX-associated genes that co-expressed with all four modeling genes were marked in red. *p < 0.05, **p < 0.01, ***p < 0.001.

First, we found 2697 co-expressed genes of 40 TEX-associated genes, crossed these genes with the DEGs between the two clusters, and screened 337 genes. Using univariate and multivariate regression analysis, 72 genes that were prognostically relevant were identified (**Figure 6A**). Subsequently, Lasso-Cox regression analysis was per-formed on these 72 genes. When the value of λ is 0.14, the model is the most stable and accurate (**Figure 6B**). Finally, a model containing four genes (PLEKHO2, VSIG4, MPP1, and GBP2) was successfully established (**Figure 6C**). The significance of these genes as prognostic indicators was confirmed through Kaplan–Meier analysis and ROC curves (**Figures 6D–K**). To further investigate the correlation between the expression of the modeled genes and TEX-associated genes, we generated co-expression heatmaps for these four genes and the 40 TEX-associated genes (**Figures 7A–D**). The heatmaps provided visual evidence of the correlation between the expression of the modeled genes and TEX-associated genes. Additionally, an upset plot was created to illustrate the intersection of TEX-associated genes co-expressed with each of the four modeled genes. The plot revealed that 13 TEX-associated genes were co-expressed with all four modeled genes (**Figure 7E**). Furthermore, we constructed a protein-protein interaction (PPI) network using the STRING database, which demonstrated the connections between these four modeled genes and the 40 TEX-associated genes at the protein level (**Figure 7F**).

### 3.5 Evaluation of prognostic signature

**Figure 8:**
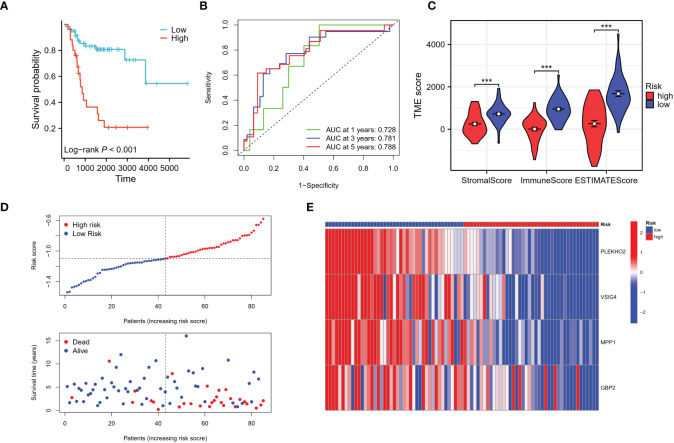
Evaluation of the risk signature. **(A)** The Kaplan-Meier analysis demonstrated that the model, based on the four genes, successfully stratified osteosarcoma patients in the TARGET training cohort into high and low-risk groups. **(B)** Additionally, time-dependent ROC curves were plotted in the training cohort, showing the area under the curve (AUC) values at different time points: 1 year (AUC = 0.728), 3 years (AUC = 0.781), and 5 years (AUC = 0.788). **(C)** The high-risk group in the training cohort displayed low immune infiltration levels, as evaluated by the Stromal score, immune score, and ESTIMATE score derived from the ESTIMATE algorithm. **(D, E)** The distribution of risk scores, survival status of each patient, and a heatmap depicting the prognostic 4-gene signature were analyzed in the TARGET cohort. ***p < 0.001.

Using the risk score of each patient in the training cohort, patients were classified into high and low-risk groups. The results showed that patients in the low-risk group had a higher overall survival rate compared to those in the high-risk group (**Figure 8A**). The predictive ability of the risk model was evaluated for 1 to 5-year survival and was found to have an area under the ROC curve (AUC) of 0.728, 0.781, and 0.788 respectively for 1, 3, and 5 years (**Figure 8B**). The differences in the immune status between the high and low-risk groups were analyzed using the ESTIMATE algorithm, and it was found that patients in the low-risk group had significantly higher stromal and immune scores, as well as ESTIMATE scores compared to those in the high-risk group (**Figure 8C**). As the risk score increased, the number of deaths increased and the survival time was significantly shorter (**Figures 8D, E**). These findings indicate that the risk model is a predictive factor for osteosarcoma prognosis and has a significant correlation with the tumor immune microenvironment (TIME) of osteosarcoma patients. Additionally, we employed the CIBERSORT algorithm to evaluate the association between immune infiltration status and these four genes alongside the risk score (see **Supplementary Figure S2**). Notably, all four genes exhibited a positive correlation with the immune infiltration of CD8 T cells, whereas the risk score displayed a negative correlation with CD8 T cells. This alignment is consistent with the observed poorer prognosis within the high-risk patient cohort.

### 3.6 Independence of the constructed risk model

Univariate and multivariate Cox regression analysis revealed that the risk score was an independent predictive marker for osteosarcoma prognosis (**Supplementary Table S1**). The risk model was further evaluated in patients with different metastasis statuses and genders, and it was found to be an effective predictor in men, women, and metastatic patients, with lower risk scores indicating a better prognosis (**Supplementary Figures S1A–D**). There was no significant difference in risk scores among patients with different genders and lesion sites, however, there was a significant difference in risk scores among patients with different metastatic statuses (**Supplementary Figures S1E–G**). These findings suggest that the risk model can serve as an independent prognostic factor for patients with osteosarcoma.

### 3.7 Validation of the prognostic signature

**Figure 9:**
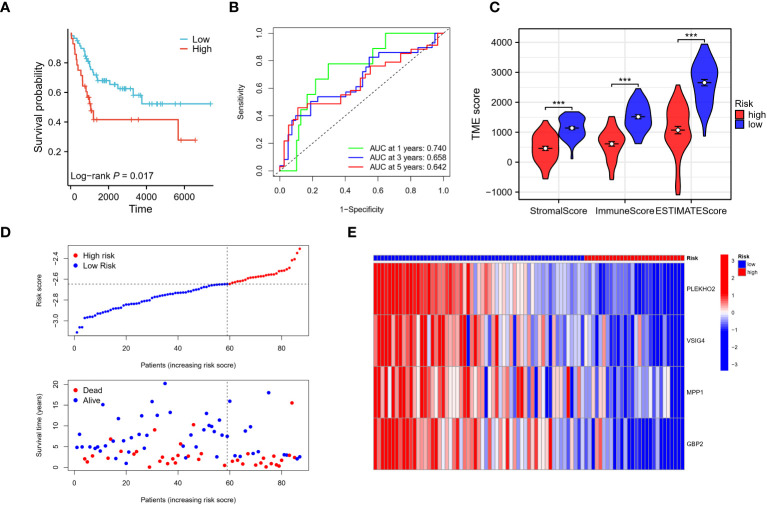
Verification of the risk signature. **(A–E)** The reliability of the model was validated in the GEO validation cohort through several analyses. Firstly, Kaplan-Meier analysis was performed to assess the model’s ability to stratify osteosarcoma patients into high and low-risk groups. Secondly, time-dependent ROC curves were generated to evaluate the predictive performance of the model at different time points. The AUC values were calculated to quantify the model’s accuracy. Finally, the stromal score, immune score, and ESTIMATE score were utilized to assess the tumor microenvironment in the validation cohort, providing additional validation of the model’s reliability. *** p < 0.001.

The patients in the validation cohort were stratified into high- and low-risk groups based on their respective risk scores. Results were comparable to those observed in the training cohort, with patients in the low-risk group exhibiting improved overall survival compared to those in the high-risk group (**Figure 9A**). Time-dependent receiver operating characteristic (ROC) curve analysis revealed an area under the curve (AUC) of 0.740, 0.658, and 0.642 for 1, 3, and 5 years, respectively (**Figure 9B**). Analysis using the ESTIMATE algorithm indicated that patients in the low-risk group had significantly higher stromal and immune scores, as well as ESTIMATE scores compared to those in the high-risk group (**Figure 9C**). Additionally, as the risk scores increased, a corresponding increase in mortality and a significant reduction in survival times were observed (**Figures 9D, E**). These results demonstrate that the risk model has predictive utility for osteosarcoma prognosis and is significantly associated with the tumor immune microenvironment (TIME) in the validation cohort.

### 3.8 Prognostic nomogram

**Figure 10:**
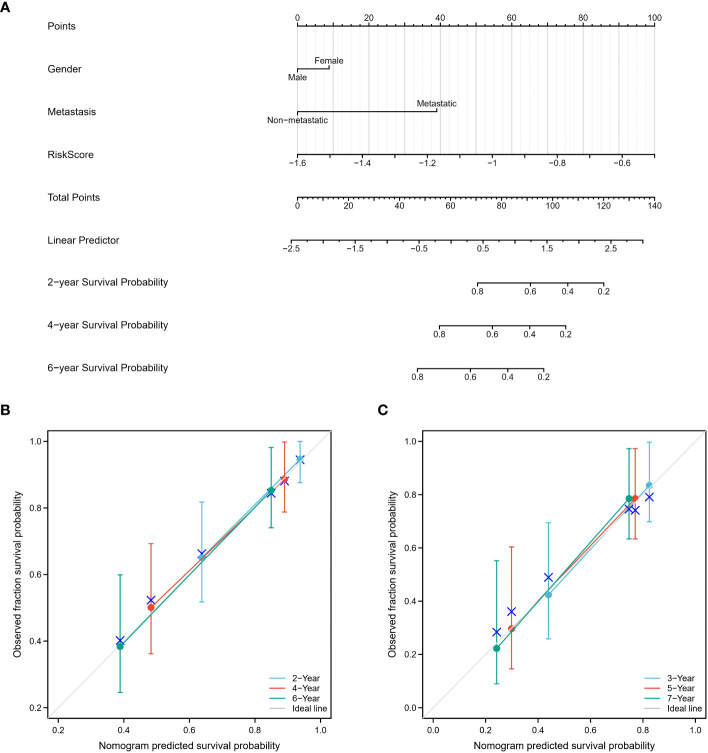
Construction and calibration of the nomogram. **(A)** A nomogram was constructed, incorporating gender, metastasis status, and risk scores, to enhance the prediction of survival outcomes. This nomogram serves as a graphical tool that combines multiple variables to estimate an individual’s survival probability. **(B, C)** The accuracy of the nomogram was assessed using both the training and validation cohorts. The observed survival rates were represented by blue, red, and green lines, while the optimized survival rate was illustrated by the gray line. The results demonstrated a favorable agreement between the observed and optimized values in both the training and validation cohorts, indicating the nomogram’s reliability in predicting survival outcomes.

A nomogram was developed that integrates gender, metastasis status, and risk scores for improved survival prediction (**Figure 10A**). Patients can obtain their respective survival rate scores based on their individual characteristics. Specifically, for each osteosarcoma patient, three points can be assigned based on their gender, metastatic status, and risk score. The sum of these three scores yields the total points. By vertically drawing a line from the corresponding point on the total points scale, it intersects with the survival probability scales at 2, 4, and 6 years, enabling the patient to determine their predicted survival probability. The accuracy of the nomogram was verified using both the training and validation cohorts, with observed survival rates depicted by blue, red, and green lines in **Figures 10B**, **C**, and the optimized survival rate represented by the gray line. Results indicate a good agreement between observed and optimized values in both the training and validation cohorts.

### 3.9 Evaluation of therapeutic responses in low-risk and high-risk groups

**Figure 11:**
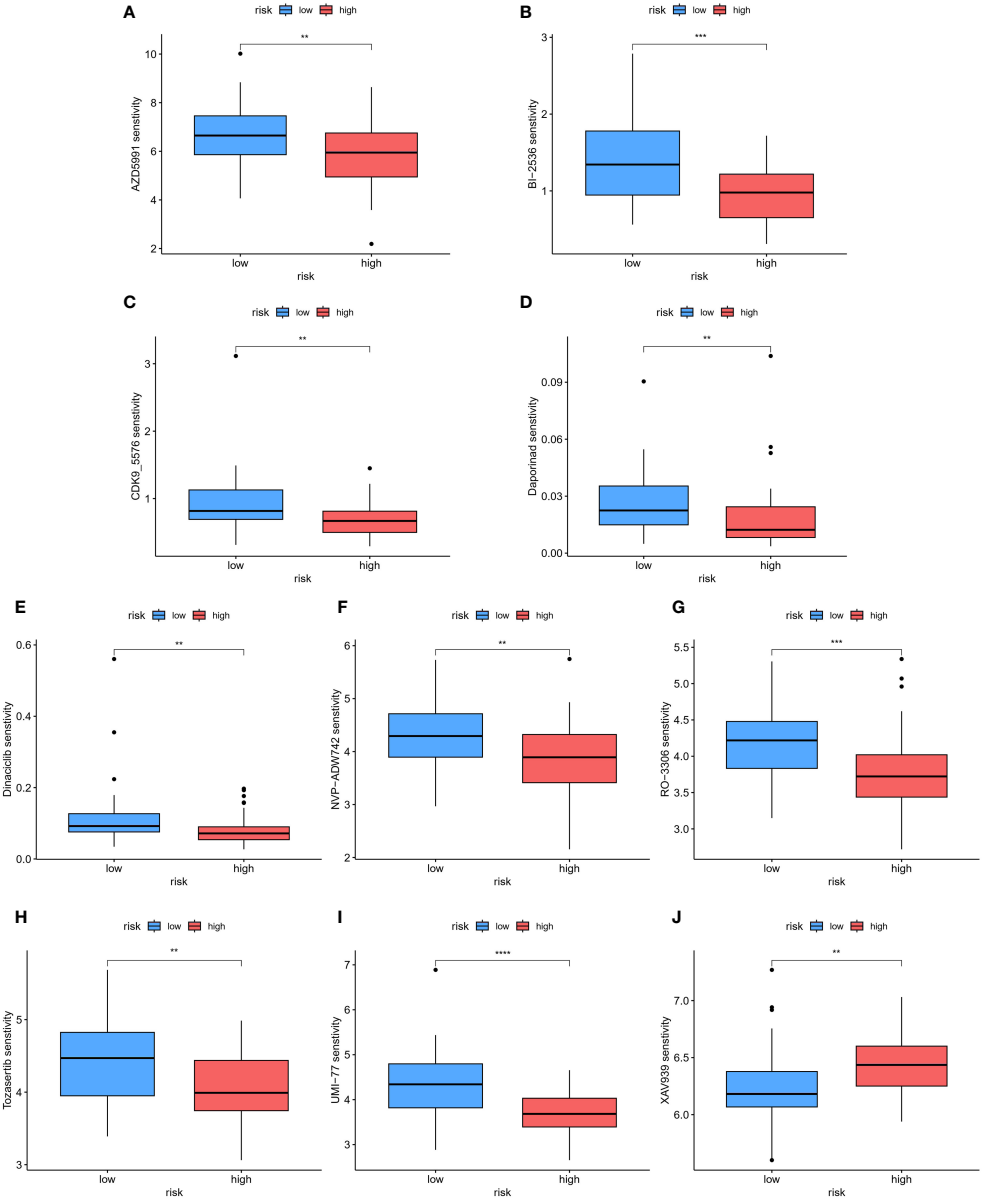
The patients in the high- and low-risk groups showed different sensitivities to various drugs. **(A–J)** Using this algorithm, 10 drugs (AZD5991, BI-2536, CDK9_5576, Dapoinad, Dinaciclib, NVP-ADW742, RO-3306, Tozasertib, UMI-77, and XAV939) were identified to have significantly different responses between the high-risk and low-risk groups. The high-risk group demonstrated higher sensitivity to 9 drugs compared to the low-risk group. The low-risk group showed higher sensitivity to XAV939. **p < 0.01; ***p < 0.001, ****p < 0.0001.

To assess the therapeutic responses in the low-risk and high-risk groups, we conducted an analysis using the oncoPredict R package to explore the drug therapy data available in the Genomics of Drug Sensitivity in Cancer (GDSC) database based on the half-maximal inhibitory concentration (IC50). With this algorithm, we identified 10 drugs, including AZD5991, BI-2536, CDK9_5576, Dapoinad, Dinaciclib, NVP-ADW742, RO-3306, Tozasertib, UMI-77, and XAV939, that exhibited significantly different responses between the high-risk and low-risk groups. Specifically, the high-risk group displayed significantly higher sensitivity to 9 drugs (**Figures 11A–I**) compared to the low-risk group, while the low-risk group exhibited higher sensitivity to XAV939 (**Figure 11J**). These findings suggest that these small molecule drugs may hold potential as therapeutic options for osteosarcoma. However, further analysis and investigation are required in future studies. Overall, these results indicate that our risk model has practical implications for guiding drug treatment selection in osteosarcoma patients.

### 3.10 ScRNA-Seq analysis in osteosarcoma

To comprehensively comprehend the distribution of model genes within the osteosarcoma tumor microenvironment (TME), we conducted an in-depth analysis of scRNA-seq data obtained from osteosarcoma patients. After stringent quality control measures, we identified a total of 179,499 cells within osteosarcoma samples, forming the basis for subsequent analyses. Through the assessment of feature gene expression, we delineated 12 major clusters within the osteosarcoma TME (**Supplementary Figure S3A**). The resulting t-SNE plot illustrated the annotation of these 12 distinct cell clusters, encompassing various cell types such as B cells (CD79A+, MS4A1+), chondroblastic osteosarcoma cells (ACAN+, COL2A1+, SOX9+), endothelial cells (PECAM1+, EGF17+, PLVAP+), fibroblast cells (COL3A1+), mast cell (MS4A2+, TPSB2+, HPGDS+, GATA2+, TPSAB1+, CPA3+, LTC4S+, RGS13+), myeloid cells (CD68+, LYZ+), neutrophil cells (CSF3R+, CXCL8+, MNDA+, S100A8+), osteoblastic osteosarcoma cells (COL1A1+, CDH11+, RUNX2+, ALPL+, IBSP+), osteoclastic cells (CTSK+, MMP9+, ACP5+), plasma cells (IGHG1+, JCHAIN+, MZB1+), proliferating osteoblastic osteosarcoma cells (PCNA+, MKI67+) and T and NK cells (CD3D+, CD8A+, CD4+, GNLY+, NKG7+), as illustrated in **Supplementary Figure S3B**.

Moreover, the authors further categorized the NK cell and T cell clusters, leading to the classification of these cells into 10 distinct clusters based on their characteristic gene expression patterns (**Supplementary Figure S4A**). These subtypes included memory CD4+ T cells (CD3G+, CD4+, S100A4+, ANXA1+, CD40LG+, CXCR6+, CXCR3+), exhausted CD4+ T cells (CD3G+, CD4+,HAVCR2+, LAG3+, TIGIT+, CzTLA4+, BTLA+), naive CD4+ T cells (CD3G+, CD4+, CCR7+, SELL+, LEF1+, TCF7+, ZNF683+), memory CD8+ T cells (CD3G+, CD8A+, S100A4+, ANXA1+, CD40LG+, CXCR6+, CXCR3+), exhausted CD8+ T cells (CD3G+, CD8A+,HAVCR2+, LAG3+, TIGIT+, CTLA4+, BTLA+), naive CD8+ T cells (CD3G+, CD8A+, CCR7+, SELL+, LEF1+, TCF7+, ZNF683+), proliferating CD8+ T cells (CD3G+, CD8A+, MKI67+, CDK1+, STMN1+), NK cells (NCR1+, TYROBP+, FCGR3A+, CX3CR1+, FGFBP2+, KLRG1+, ZEB2, TRGC2+, XCL1+, XCL2+), mucosal-associated invariant T cells (MAIT, CD3G+, SLC4A10+, NCR3, ZBTB16, KLRB1), and stress response state T cells (CD3G+, HSPA6+, DNAJB1+, HSPH1+, HSP90AA1+, BAG3+, HSPD1+, HSPE1+, HSPB1+, SERPINH1+, ZFAND2A+), as shown in **Supplementary Figure S4B**.

Furthermore, to validate the precision of our clustering, we utilized the AUCell algorithm, focusing on T cell exhaustion-related gene sets sourced from the GSEA database. The resulting UMAP plot showcased relatively higher T cell exhaustion gene AUCell scores within the annotated CD4+ and CD8+ exhausted T cell clusters compared to other identified clusters (**Supplementary Figure S4C**). Correspondingly, the UMAP plot and violin plots (**Supplementary Figures S4D, E**) emphasized that our risk gene GBP2 exhibited heightened expression in CD8+ proliferative T cells, exhausted CD8+ T cells, and exhausted CD4+ T cells, with minimal expression detected in stress response state (TSTR) cells. Additionally, genes associated with cell proliferation, such as PCNA and MKI67, displayed elevated expression specifically within CD8+ proliferative T cells.

### 3.11 Validation of the prognostic impact of the four genes’ expression

**Figure 12:**
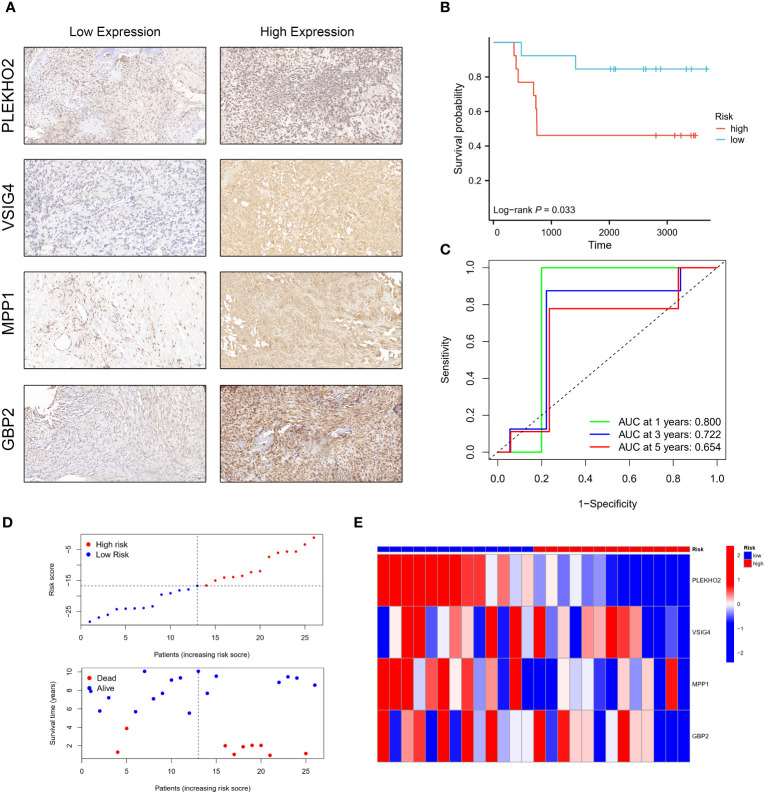
Validation of the prognostic model using the H-score obtained by immunohistochemistry (IHC). **(A)** Osteosarcoma patients were categorized into low and high-risk groups based on the immunohistochemistry (IHC) results of PLEKHO2, VSIG4, MPP1, and GBP2. **(B)** The high-risk group exhibited a poorer survival outcome compared to the low-risk group. **(C)** Time-dependent ROC curves were generated in the IHC validation cohort, with AUC values of 0.800 at 1 year, 0.722 at 3 years, and 0.654 at 5 years. **(D, E)** The distribution of risk scores, the survival status of each patient, and a heatmap depicting the prognostic 4-gene signature were analyzed in the IHC validation cohort.

To validate the prognostic impact of our four-gene expression model at the protein level, we obtained paraffin tissue sections from 26 osteosarcoma patients. Immunohistochemistry (IHC) experiments were conducted to assess the expression levels of the four modeling genes: PLEKHO2, GBP2, MPP1, and VSIG4 (**Figure 12A**). The H-score was determined for each gene in each patient, and these scores were used to calculate a risk score using the risk score formula. Based on their risk scores, patients were categorized into high-risk and low-risk groups. Kaplan-Meier (KM) analysis demonstrated that patients in the high-risk group had significantly worse prognoses compared to those in the low-risk group (**Figure 12B**). This finding is consistent with the results obtained from the TARGET and GEO cohorts. Time-dependent ROC curve analysis showed AUCs of 0.800, 0.722, and 0.654 for 1, 3, and 5 years, respectively (**Figure 12C**). Furthermore, as the risk scores increased, there was a corresponding increase in mortality and a significant decrease in survival times (**Figures 12D, E**). These results provide further confirmation of the prognostic significance of our risk model at the protein level.

### 3.12 The downregulation of GBP2 promoted the proliferation, migration, and invasion of osteosarcoma cells and inhibited apoptosis

**Figure 13:**
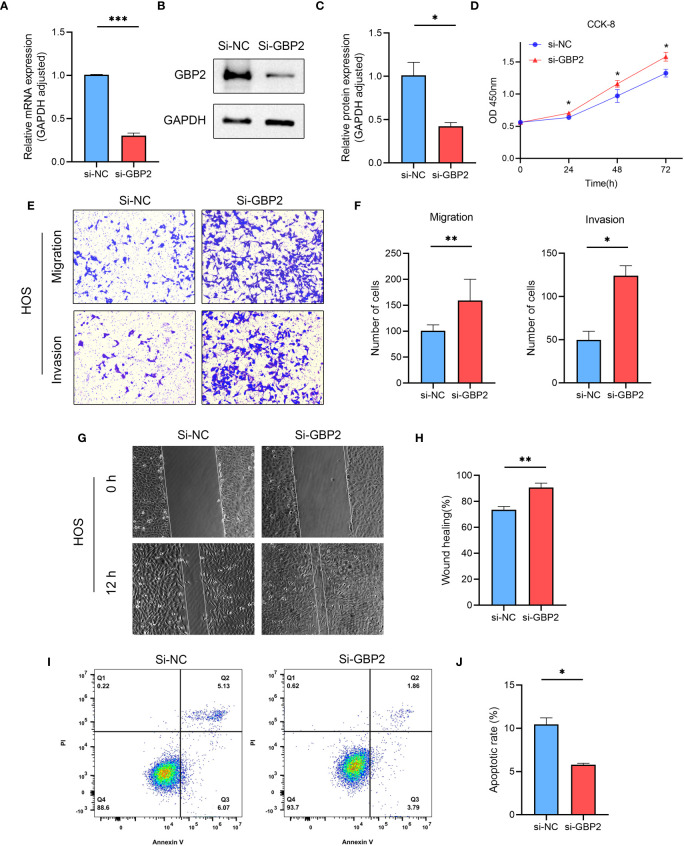
The effect of GBP2 on osteosarcoma cell line HOS proliferation, migration, invasion and apoptosis. **(A–C)** The expression of GBP2 was downregulated using siRNA, as confirmed by Western blot and RT-qPCR analysis. **(D)** CCK-8 assays demonstrated that the downregulation of GBP2 led to increased cell proliferation in osteosarcoma cells. **(E, F)** Transwell assays showed that GBP2 downregulation significantly enhanced cell migration and invasion in osteosarcoma cells. **(G, H)** Wound healing assays also indicated that GBP2 downregulation markedly promoted cell migration ability in osteosarcoma cells. **(I, J)** Flow cytometry analysis revealed that GBP2 downregulation inhibited apoptosis in osteosarcoma cells. *p < 0.05, **p < 0.01, ***p < 0.001.

**Figure 14:**
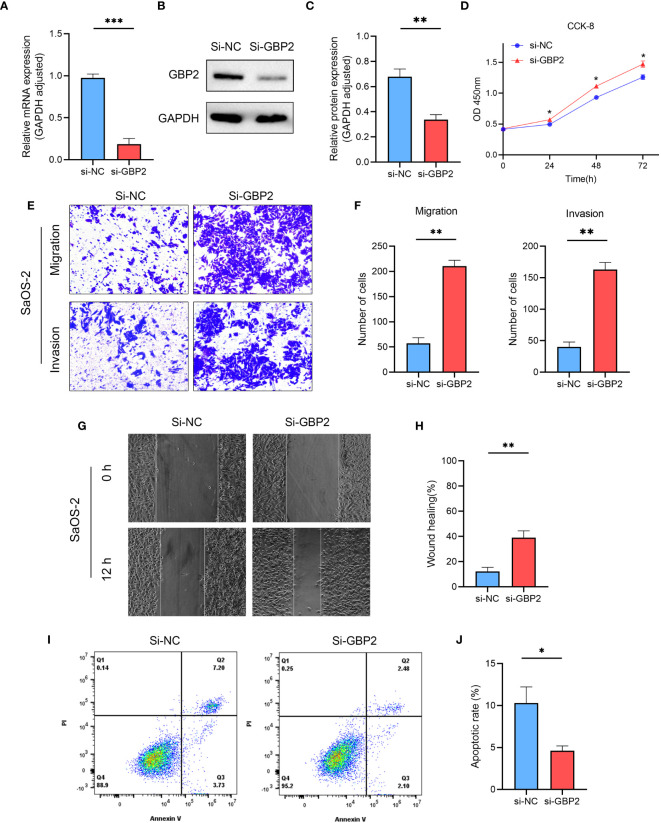
The effect of GBP2 on osteosarcoma cell line SaOS-2 proliferation, migration, invasion and apoptosis. **(A–C)** The expression of GBP2 was downregulated using siRNA, as confirmed by Western blot and RT-qPCR analysis. **(D)** CCK-8 assays demonstrated that the downregulation of GBP2 led to increased cell proliferation in osteosarcoma cells. **(E, F)** Transwell assays showed that GBP2 downregulation significantly enhanced cell migration and invasion in osteosarcoma cells. **(G, H)** Wound healing assays also indicated that GBP2 downregulation markedly promoted cell migration ability in osteosarcoma cells. **(I, J)** Flow cytometry analysis revealed that GBP2 down-regulation inhibited apoptosis in osteosarcoma cells. *p < 0.05, **p < 0.01, ***p < 0.001.

We employed siRNA to downregulate GBP2 expression (**Figures 13A–C**) and investigated its impact on cell proliferation, migration, invasion, and apoptosis. CCK-8 assays revealed that the downregulation of GBP2 resulted in increased cell proliferation in HOS cells (**Figure 13D**). Transwell assays demonstrated that GBP2 downregulation significantly enhanced cell migration and invasion in HOS cells (**Figures 13E, F**). Wound healing assays also indicated that GBP2 downregulation markedly promoted cell migration ability in HOS cells (**Figures 13G, H**). Moreover, flow cytometry analysis revealed that GBP2 downregulation inhibited apoptosis in HOS cells (**Figures 13I, J**). The afore-mentioned findings were corroborated by an independent cell line (SaOS-2) (**Figures 14A–J**). Collectively, these findings indicate that the downregulation of GBP2 gene promotes cell proliferation, migration, and invasion in HOS cells and SaOS-2 cells while suppressing apoptosis.

## 4 Discussion

The tumor microenvironment is a complex ecosystem comprising various cellular and molecular components that collectively influence tumor growth and development, including immune checkpoint-related molecules (20). Recent research has emphasized the crucial role of the immune microenvironment within the tumor microenvironment, highlighting the need for a comprehensive understanding for effective immunotherapy. Our study identified significant differences in the immune microenvironment between two analyzed subgroups: the low-risk osteosarcoma group (Cluster 1) and the high-risk osteosarcoma group (Cluster 2). Patients in the low-risk group exhibited a superior overall survival rate compared to those in the high-risk group. Deeper analysis revealed a pronounced upregulation of CD4+ T cells, CD8+ T cells, dendritic cells, and macrophages in the low-risk group, indicating an active and enhanced immune response within their tumor microenvironment. In contrast, the high-risk group showed immune system inhibition, characterized by lower expression levels of these immune cell populations.

Further analysis of gene ontology, KEGG pathways, and gene set enrichment revealed the significant suppression of crucial signaling pathways involved in T cell receptor signaling, Toll-like receptor (TLR) signaling, B cell receptor signaling, antigen presentation, as well as ferroptosis, pyroptosis, necroptosis, and immunogenic cell death in the high-risk osteosarcoma group. The inhibition of T cell receptor signaling suggests compromised activation and functionality of T cells, which are essential for effective anti-tumor immune responses. Additionally, the suppression of TLR signaling implies a diminished ability of immune cells to detect and respond to pathogenic signals within the tumor microenvironment. The inhibition of antigen presentation signifies a potential evasion mechanism employed by osteosarcoma cells to evade immune surveillance. Moreover, the suppression of ferroptosis, pyroptosis, necroptosis, and immunogenic cell death in the high-risk group suggests the disruption of multiple forms of cell death that can have important implications for tumor development. These cellular death pathways often play critical roles in eliminating cancer cells and triggering immune responses against tumors. Their suppression may contribute to tumor immune escape and progression.

Collectively, the identified inhibition of T cell receptor signaling, TLR signaling, B cell receptor signaling, antigen presentation, and the suppression of ferroptosis, pyroptsis, necroptosis, and immunogenic cell death are closely associated with T cell exhaustion in osteosarcoma. These intricate interactions form a complex network of immune evasion mechanisms that limit the immune system’s ability to effectively target and eliminate tumors, ultimately fostering tumor development and progression. Advancing our understanding of these interactions can provide valuable insights into the mechanisms of tumor immune evasion and facilitate the development of novel therapeutic strategies to overcome T cell exhaustion and enhance anti-tumor immune responses.

To elucidate key target genes, we developed a prognostic model based on T-cell exhaustion-associated genes in osteosarcoma. Notably, our analysis identified a significant correlation between the prognosis of osteosarcoma patients and four genes: V-set and Immunoglobulin Domain-containing Protein 4 (VSIG4), Membrane Palmitoylated Protein 1 (MPP1), Pleckstrin Homology-domain-containing family O member 2 (PLEKHO2), and Guanylate Binding Protein 2 (GBP2). Subsequently, we validated the model using our osteosarcoma patient cohort. Kaplan-Meier analysis demonstrated that individuals classified as high-risk according to our model experienced significantly worse prognoses compared to those classified as low-risk. Furthermore, time-dependent ROC curve analysis supported the prognostic utility of our risk model. These findings were consistent with outcomes observed in independent TARGET and GEO cohorts, providing compelling evidence for the prognostic value of the four-gene expression model in osteosarcoma. By incorporating key genes associated with T-cell exhaustion, our model offers improved predictive accuracy for patient outcomes.

Although PLEKHO2, VSIG4, MPP1, and GBP2 have been implicated in the pathogenesis of several cancers, their roles in osteosarcoma have not been reported to date. VSIG4, a type 1 transmembrane protein of the B7-related immunoglobulin superfamily, is normally expressed on tissue-resident macrophages (21, 22). It has been shown to inhibit the production of IL-2, the proliferation of effector CD8+ T cells, and induce regulatory T cells (Tregs) (23). The correlation between VSIG4 expression and prognosis in various tumors has been inconsistent across different studies. For example, overexpression of VSIG4 in high-grade gliomas (24), gastric cancer (25), and multiple myeloma (26) have been associated with poor prognosis, while low VSIG4 expression in tumor tissue was related to poor prognosis in patients with HBV-associated hepatocellular carcinoma (27).

MPP1, also known as p55, is a member of the membrane-associated guanylate kinase homologues family of signaling proteins. Initially identified as a scaffolding protein in erythrocytes, MPP1 forms a tripartite complex with protein 4.1R and glycophorin C, which helps to stabilize the actin cytoskeleton and its association with the plasma membrane (28, 29). Moreover, MPP1 has been found to regulate the polarity of neutrophils (30). In the context of neurofibromatosis type 2, MPP1 can bind to the FERM structural domain of the NF2 protein, a tumor suppressor gene encoded by the NF2 gene, potentially leading to a tumor-suppressive effect (31).

PLEKHO2, a member of the PH-domain-containing protein superfamily, was found by Zhang et al. to play a critical role in immune function and cell death. In their studies, the absence of PLEKHO2 in mice resulted in significantly reduced numbers of macrophages, and macrophages with PLEKHO2 deficiencies produced more apoptosis and caspase-3 activity (32). Additionally, PLEKHO2 was found to inhibit TNFα-induced cell death by suppressing the activation of receptor interaction protein kinase 1 (33).

GBP2 is a member of the p65 Guanyline Binding Protein (GBP) family. A study by Godoy et al. found that high expression of GBP2 in breast cancer was correlated with a better prognosis and a robust T-cell response (34). On the other hand, low expression of GBP2 in patients with microsatellite stable colorectal cancer was associated with poor prognosis and increased metastasis. More importantly, the knockout of GBP2 resulted in a reduction in CD8+ T cell infiltration and blunted the efficacy of PD-1 blockade in tumor-bearing mice (35). Zhang et al. analyzed the role of GBP2 in skin cutaneous melanoma using data from the TCGA database and found that GBP2 expression was positively correlated with infiltration by B-cells, CD8+ T-cells, CD4+ T-cells, macrophages, neutrophils, and dendritic cells. Furthermore, higher levels of GBP2 expression were found in patients with metastatic skin cutaneous melanoma, as well as in patients with better survival rates (36). Another study by Geng et al. demonstrated the anti-tumor effects of GBP2, which were achieved through the inhibition of the Wnt/β-catenin pathway in skin cutaneous melanoma (37). Although the involvement of Guanylate Binding Protein 2 (GBP2) has been well established in the pathogenesis of various tumor types, its specific function in osteosarcoma remains largely unexplored. Thus, we conducted an in-depth investigation into the role of GBP2 in osteosarcoma.

Our experimental results revealed that the downregulation of GBP2 enhanced cell proliferation, as demonstrated by increased cell viability observed in CCK-8 assays. This finding suggests that GBP2 may exert a suppressive effect on cell growth in osteosarcoma. Furthermore, the downregulation of GBP2 had a profound impact on cell migration and invasion, as indicated by the enhanced migratory and invasive abilities observed in Transwell assays. Additionally, wound healing assays also confirmed the promotion of cell migration ability upon GBP2 downregulation. These findings collectively suggest that GBP2 may play a role in restraining the migratory and invasive potential of HOS cells and SaOS-2 cells in osteosarcoma. Moreover, flow cytometry analysis demonstrated that GBP2 downregulation resulted in the inhibition of apoptosis in HOS cells and SaOS-2 cells. This suggests that GBP2 may possess pro-apoptotic properties in osteosarcoma, and its downregulation may confer a survival advantage to the cancer cells. These results indicate that GBP2 may function as a potential tumor suppressor in osteosarcoma, and further investigations are warranted to elucidate the underlying molecular mechanisms by which GBP2 modulates these cellular processes. Understanding the precise role of GBP2 in osteosarcoma could potentially pave the way for the development of novel therapeutic strategies targeting this gene to inhibit tumor progression and improve patient outcomes.

Meanwhile, our study also identified 10 drugs, including AZD5991, BI-2536, CDK9_5576, Dapoinad, Dinaciclib, NVP-ADW742, RO-3306, Tozasertib, UMI-77, and XAV939, that exhibited significantly different responses between the high-risk and low-risk osteosarcoma groups. Notably, the high-risk group displayed significantly higher sensitivity to 9 of these drugs compared to the low-risk group, while the low-risk group exhibited higher sensitivity to XAV939. These findings suggest that these small-molecule drugs may hold potential as therapeutic options for osteosarcoma, with their efficacy varying based on the risk profile of the patients. Further exploration of these drugs and their mechanisms of action in osteosarcoma could provide valuable insights for developing novel therapeutic strategies to inhibit tumor progression and improve patient outcomes.

However, this study has some limitations that need to be addressed. Firstly, while our bioinformatics analysis has indeed revealed a positive correlation between the expression of the four genes (PLEKHO2, VSIG4, MPP1, and GBP2) and the infiltration of CD8+ T cells in osteosarcoma, the precise mechanism by which these genes foster CD8+ T cell infiltration remains elusive. Wang et al. similarly observed a positive correlation between GBP2 and CD8+ T cell infiltration in colorectal cancer (34). Their experiments demonstrated that the genetic knockout of GBP2 resulted in diminished migration of CD8+ T cells, whereas supplementation with CXCL10/11 restored T cell migration. Hence, they propose that the heightened infiltration of CD8+ T cells might be ascribed to the upregulation of CXCL10/11 facilitated by GBP2. Secondly, single-cell RNA-seq analysis provides a profound understanding of intra-tumoral diversity, facilitating the identification and characterization of distinct tumor subtypes. This approach unveils novel perspectives for devising more effective treatment strategies. For example, Lin and Chai et al. classified melanoma into seven subgroups using single-cell sequencing, revealing a significant finding that C4 melanoma CORO1A might be more sensitive to NK and T cells, whereas other subtypes of melanoma may exhibit higher resistance to NK and T cells (38). Therefore, we conducted single-cell RNA-seq analysis of GBP2 expression in osteosarcoma. Although the single-cell RNA-seq analysis unveiled heightened expression of GBP2 in CD8+ proliferative T cells, as well as exhausted CD4+ and CD8+ T cells, the precise causal relationship and biological implications of GBP2 in governing the proliferation and exhaustion of CD4+ and CD8+ T cells remain ambiguous, demanding further exploration in forthcoming studies. Third, throughout our validation process, inherent limitations within bioinformatics analysis arose, notably stemming from the utilization of diverse sequencing platforms across various databases, consequently introducing inherent biases. To counter these challenges, we employed a multi-faceted approach, incorporating multiple datasets obtained from both the TARGET and GEO databases, alongside data derived from our own cohort of osteosarcoma patients. This concerted effort aimed to fortify the stability of our bioinformatics analysis through data integration. Finally, we focused on investigating the role of GBP2 in osteosarcoma and did not extensively explore the functions of PLEKHO2, VSIG4, and MPP1. Future cellular or animal experiments are necessary to investigate the functional mechanisms of these genes in osteosarcoma.

## Data availability statement

Publicly available datasets were analyzed in this study. This data can be found here: TARGET repository (https://portal.gdc.cancer.gov/projects/TARGET-OS) and the Gene Expression Omnibus (https://ftp.ncbi.nlm.nih.gov/geo/series/GSE16nnn/GSE16091/matrix/; https://ftp.ncbi.nlm.nih.gov/geo/series/GSE21nnn/GSE21257/matrix/).

## Ethics statement

The studies involving humans were approved by Ethics Committee of The Second Affiliated Hospital of Nanchang University. The studies were conducted in accordance with the local legislation and institutional requirements. Written informed consent for participation in this study was provided by the participants’ legal guardians/next of kin.

## Author contributions

JZ: Visualization, Writing – original draft, Data curation, Formal Analysis, Software. JY: Writing – original draft, Methodology, Visualization. SA: Conceptualization, Methodology. DZ: Visualization, Writing – original draft. XL: Writing – review & editing, Project administration. JJ: Project administration, Supervision, Writing – review & editing.
