# Supplementary figures and images for: Exploring the immune microenvironment of osteosarcoma through T cell exhaustion-associated gene expression: a study on prognosis prediction
Source: Front Immunol. 2023 Dec 15;14:1265098. doi: 10.3389/fimmu.2023.1265098 (PMC10758463; doi:10.3389/fimmu.2023.1265098)

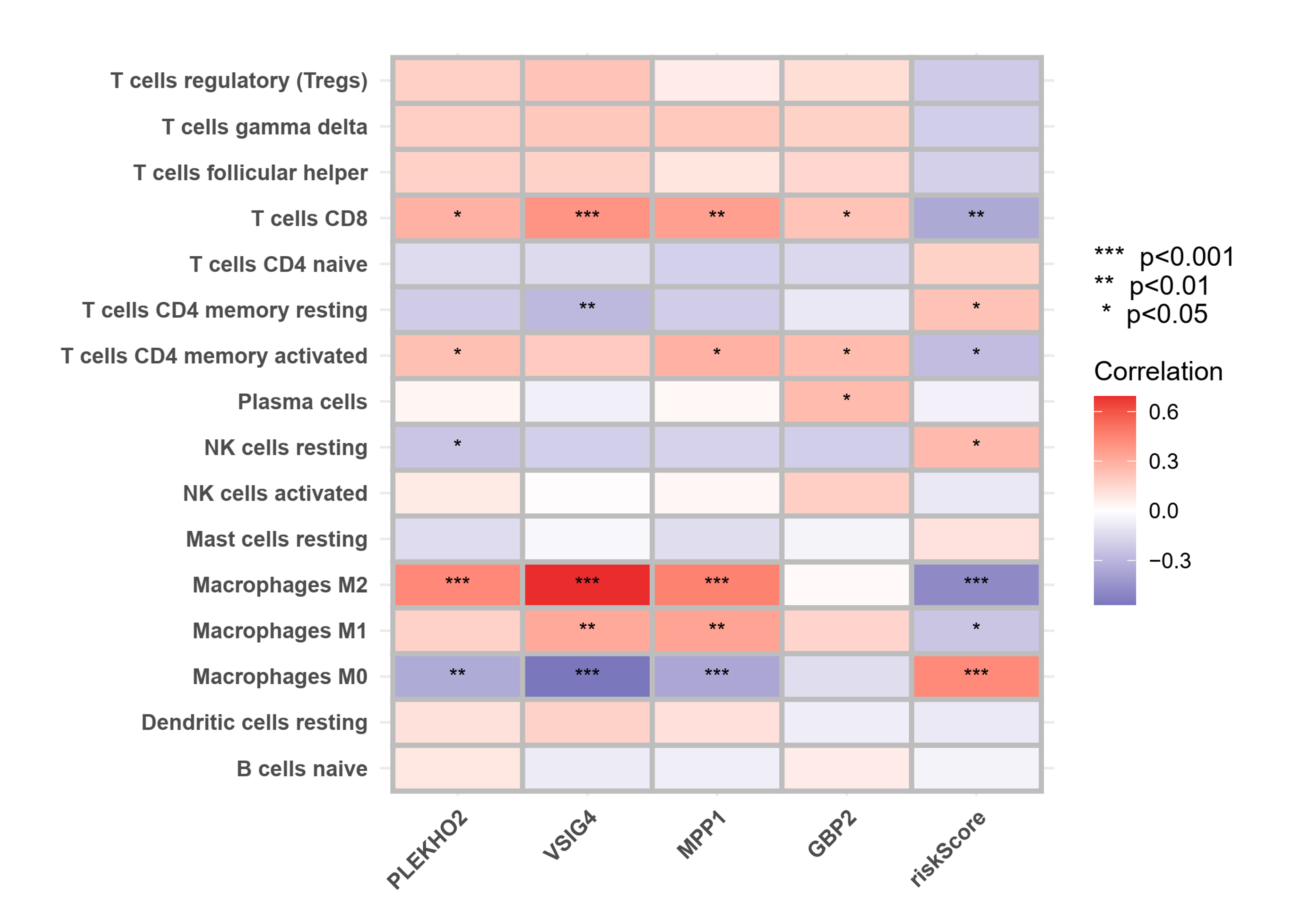

Supplement: Supplementary file 1 [file Image_1.jpeg]

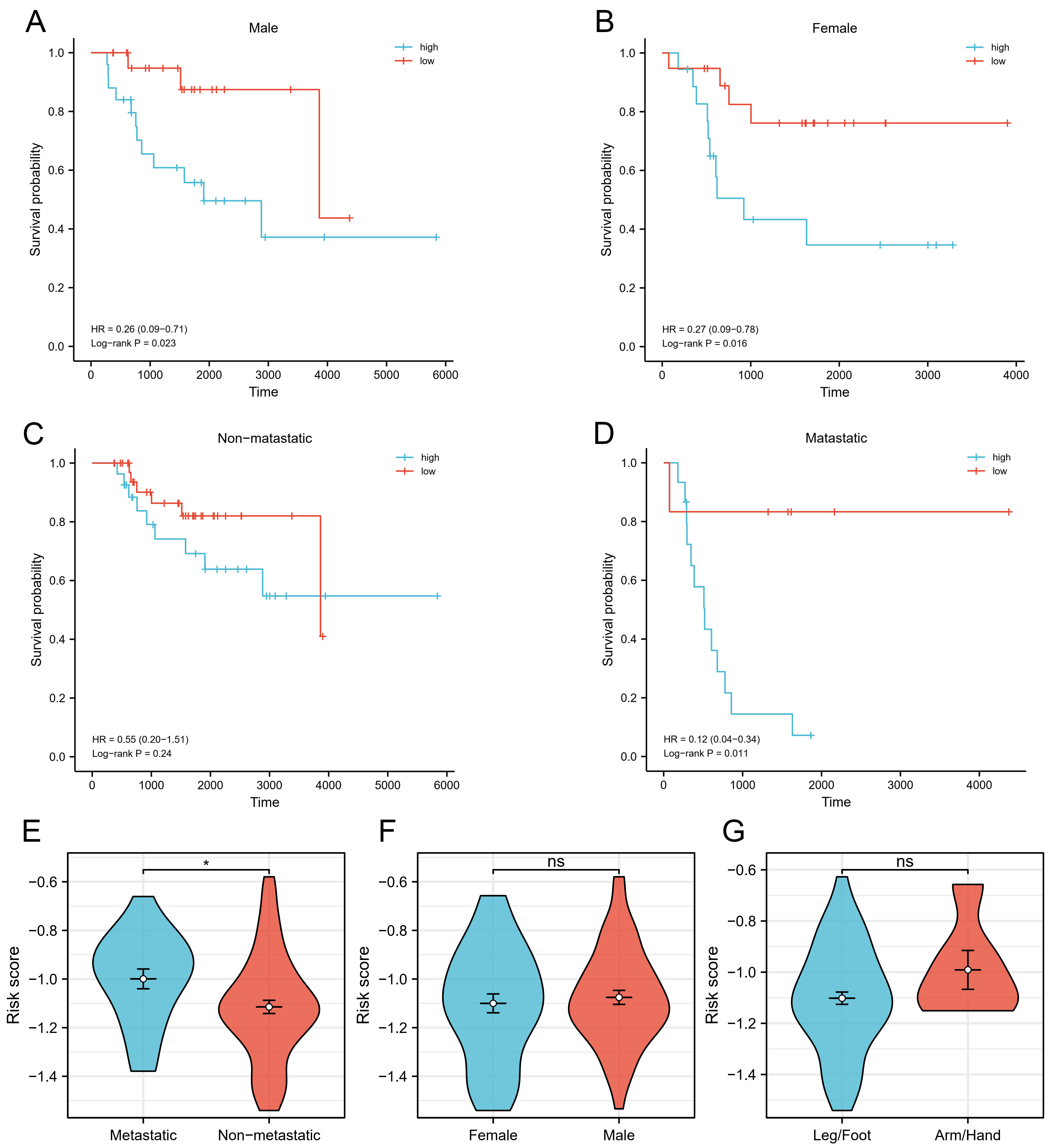

Supplement: Supplementary file 2 [file Image_2.jpeg]

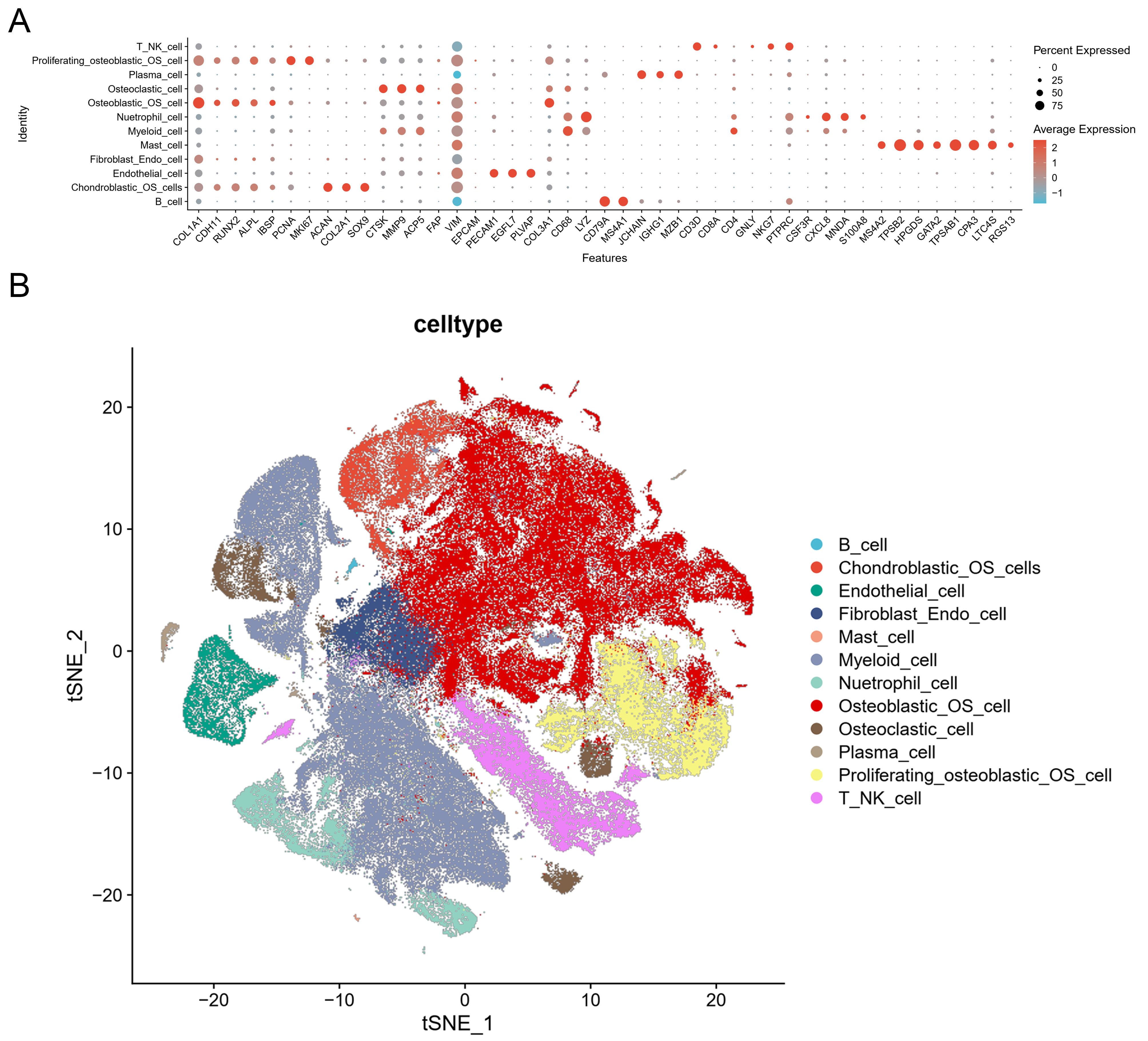

Supplement: Supplementary file 3 [file Image_3.jpeg]

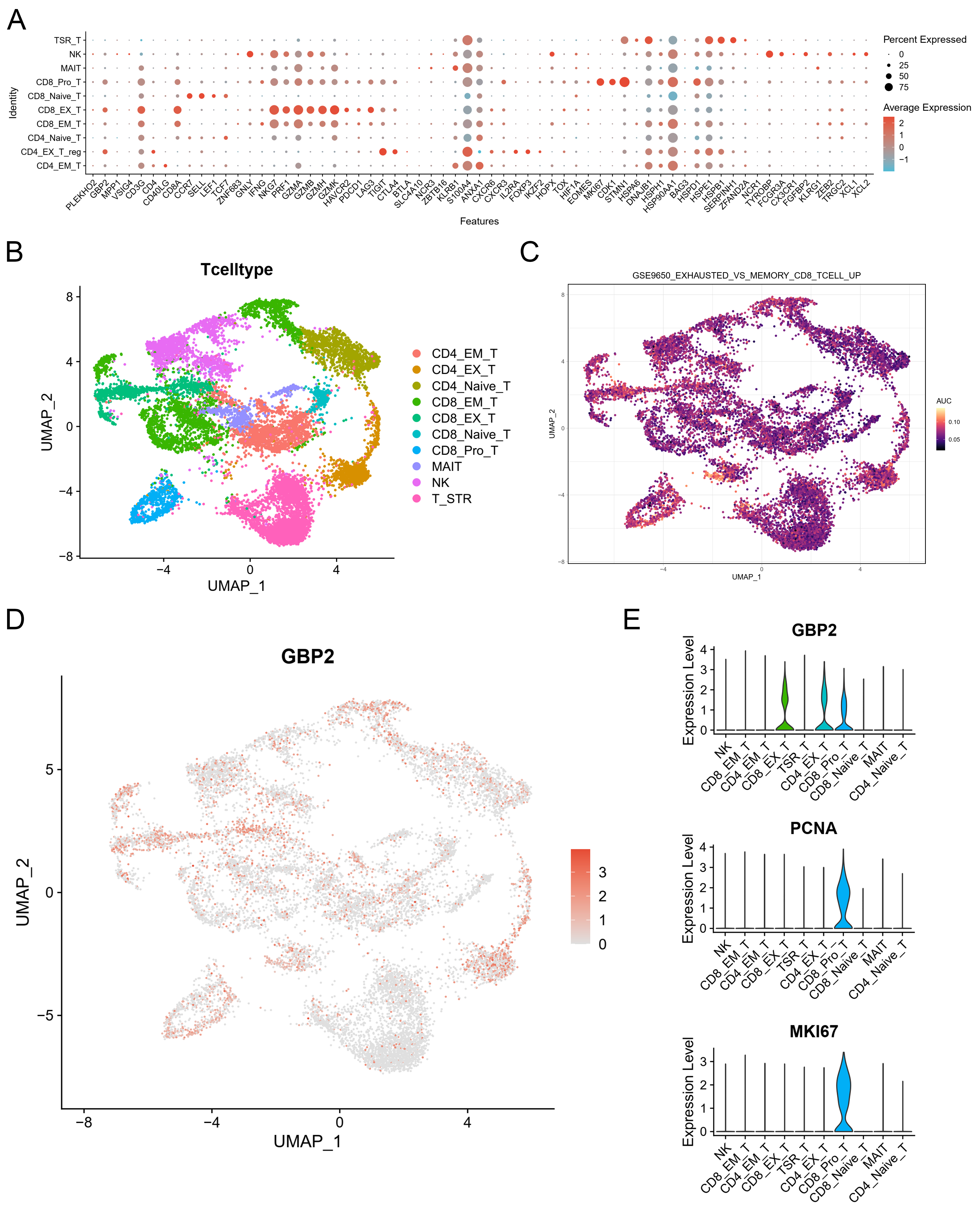

Supplement: Supplementary file 4 [file Image_4.jpeg]
