# Supplementary material for: Exploring the immune microenvironment of osteosarcoma through T cell exhaustion-associated gene expression: a study on prognosis prediction
Source: Front Immunol. 2023 Dec 15;14:1265098. doi: 10.3389/fimmu.2023.1265098 (PMC10758463; doi:10.3389/fimmu.2023.1265098)
Supplement: Supplementary file 5 [file Table_1.docx]

**Supplementary Table 1.** Univariate and multivariate Cox analyses evaluate the independent prognostic value of risk signature in osteosarcoma patients.

| **Characteristics** | **Total(N)** | **Univariate analysis** | |  | **Multivariate analysis** | |
| --- | --- | --- | --- | --- | --- | --- |
|  |  | **Hazard ratio (95% CI)** | **P value** |  | **Hazard ratio (95% CI)** | **P value** |
| Gender | 85 |  |  |  |  |  |
| Female | 37 | Reference |  |  |  |  |
| Male | 48 | 0.681 (0.328-1.416) | 0.304 |  |  |  |
| Metastasis | 85 |  |  |  |  |  |
| Metastatic | 21 | Reference |  |  |  |  |
| Non-metastatic | 64 | 0.210 (0.100-0.438) | **<0.001** |  | 0.208 (0.098-0.442) | **<0.001** |
| Risk score | 85 | 31.288 (5.236-186.973) | **<0.001** |  | 38.822 (5.433-277.427) | **<0.001** |
